# Data on the relation between renal biomarkers and measured glomerular filtration rate

**DOI:** 10.1016/j.dib.2017.08.034

**Published:** 2017-09-01

**Authors:** Hans Pottel, Laurence Dubourg, Elke Schaeffner, Bjørn Odvar Eriksen, Toralf Melsom, Edmund J. Lamb, Andrew D. Rule, Stephen T. Turner, Richard J. Glassock, Vandréa De Souza, Luciano Selistre, Karolien Goffin, Steven Pauwels, Christophe Mariat, Martin Flamant, Sebastjan Bevc, Pierre Delanaye, Natalie Ebert

**Affiliations:** a Department of Public Health and Primary Care, KU Leuven Campus Kulak Kortrijk, Kortrijk, Belgium; b Exploration Fonctionnelle Rénale, Groupement Hospitalier Edouard Herriot, Hospices Civils de Lyon, Lyon, France; c Charité University Hospital, Institute of Public Health, Berlin, Germany; d Metabolic and Renal Research Group, UiT The Arctic University of Norway, Tromsø, Norway; e Clinical Biochemistry, East Kent Hospitals University NHS Foundation Trust, Canterbury, Kent, United Kingdom; f Division of Nephrology and Hypertension, Mayo Clinic, Rochester, MN, USA; g Emeritus Professor of Medicine, Geffen School of Medicine at UCLA, Laguna Niguel, CA, USA; h Universidade de Caxias do Sul - Programa de Pós Graduação em Ciências da Saúde, Brazil; i Pontifícia Universidade Católica do Rio Grande do Sul, Porto Alegre, Brazil; j Department of Nuclear Medicine & Molecular Imaging, University Hospital Leuven, Leuven, Belgium; k Department of Cardiovascular Sciences, Department of Laboratory Medicine, University Hospital Leuven, Leuven, Belgium; l Service de Néphrologie, Dialyse et Transplantation Rénale, Hôpital Nord, CHU de Saint-Etienne, France; m Department of Renal Physiology, Hôpital Bichat, AP-HP and Paris Diderot University, Paris, France; n University Medical Centre Maribor, Clinic for Internal Medicine, Department of Nephrology, Maribor, Slovenia; o Nephrology-Dialysis-Transplantation, University of Liège, CHU Sart Tilman, Liège, Belgium

**Keywords:** Serum creatinine, Serum cystatin C, Measured glomerular filtration rate

## Abstract

The data presented in this article are related to the research article entitled “The Diagnostic Value of Rescaled Renal Biomarkers Serum Creatinine and Serum Cystatin C and their Relation with Measured Glomerular Filtration Rate” (Pottel et al. (2017) [1]). Data are presented demonstrating the rationale for the normalization or rescaling of serum cystatin C, equivalent to the rescaling of serum creatinine. Rescaling biomarkers brings them to a notionally common scale with reference interval [0.67–1.33]. This article illustrates the correlation between rescaled biomarkers serum creatinine and serum cystatin C by plotting them in a 2-dimensional graph. The diagnostic value in terms of sensitivity and specificity with measured Glomerular Filtration Rate as the reference method is calculated per age-decade for both rescaled biomarkers. Finally, the interchangeability between detecting impaired kidney function from renal biomarkers and from the Full Age Spectrum FAS-estimating GFR-equation and measured GFR using a fixed and an age-dependent threshold is shown.

**Specifications Table**

**Table:** 

Subject area	*Renal Physiology*
More specific subject area	*Renal biomarkers serum creatinine (Scr) and serum cystatin C (ScysC) and their relation with directly measured glomerular filtration rate (mGFR)*
Type of data	*Assay results for serum creatinine, serum cystatin C and directly measured glomerular filtration rate from various reference methods, demographic data*
How data was acquired	*Diagnostic assays, accepted reference methods for GFR*
Data format	*Data are presented in graphs and tables in analyzed format*
Experimental factors	*All biomarker assays are calibrated against the international standard or gold standard method (IDMS for Scr). All methods for GFR are reference methods with accepted sufficient accuracy.*
Experimental features	*See Table 1 in reference*[1].
Data source location	*See Table 2 in reference*[1]*. All data cohorts were presented in previous studies.*
Data accessibility	*The data used in this article are obtained by pooling different cohorts which are not available in a public repository, and were received by the mentioned institutes for the purpose of this study. The data from the CRIC Study reported here were supplied by the NIDDK Central Repositories.*[1]*The data are presented in summary tables and graphs within this article.*

**Value of the data**

• The data present the rationale for the choice of the rescaling factor for serum cystatin C.
• Rescaling brings the biomarker to a notionally common scale making its interpretation easy with reference to the reference interval [0.67–1.33].
• The upper limit of the reference interval (1.33) is used as a threshold to detect impaired kidney function and this is compared to the definition of impaired kidney function based on a fixed and age-dependent threshold for GFR.
• These data give new insights into the relation between renal biomarkers and measured GFR.

## 1 Data

### 1.1 Rationale for the rescaling of serum cystatin C (ScysC)

Analogous to the normalization or rescaling of serum creatinine (Scr), the normalization or rescaling factor(s) for ScysC is defined as the mean (or median) of the ScysC-distribution(s) for healthy subjects. The rescaling factors have previously been defined as Q_cysC_ = 0.82 mg/L for subjects aged < 70 years and Q_cysC_ = 0.95 mg/L for subjects aged ≥ 70 years [2]. In this article, data and a new analysis are presented to further support these choices for the rescaling of ScysC.

Only ‘healthy’ subjects were selected, that is, a subgroup is selected from the total collection of 8584 subjects, obtained from the normal population and from nephrology clinics. First, it was required that Scr/Q_crea_ ≤ 1.33, or, only subjects with ‘normal’ Scr-values were selected. Q_crea_-values for Scr have been reported for children and adolescents [3], [4]. For adults, Q_crea_ = 0.70 mg/dL is used for females and Q_crea_ = 0.90 mg/dL for males. This selection requirement reduces the total dataset from 8584 to 5352 patients. The additional requirement that mGFR ≥ 60 mL/min/1.73 m² further reduces the dataset from 5352 to 4907. Table 1 shows the numbers, mean, median, standard deviation (SD) and interquartile range (IQR) per age-decade for ScysC in this healthy subjects subgroup.

**Table 1:** Serum cystatin C concentrations for subjects with Scr/Q_crea_ ≤ 1.33 and mGFR ≥ 60 mL/min/173 m².

Age Group	n	mean	median	SD	IQR
2–10	170	0.94	0.92	0.18	0.24
10–20	352	0.96	0.93	0.22	0.29
20–30	122	0.84	0.81	0.17	0.18
30–40	293	0.79	0.78	0.14	0.16
40–50	432	0.81	0.80	0.16	0.21
50–60	1543	0.76	0.74	0.15	0.17
60–70	1317	0.81	0.78	0.16	0.19
70–80	528	0.89	0.88	0.15	0.19
80–90	147	0.96	0.96	0.14	0.19
> 90	3	1.04	1.01	0.06	0.11
	4907				

**Fig. 1:**
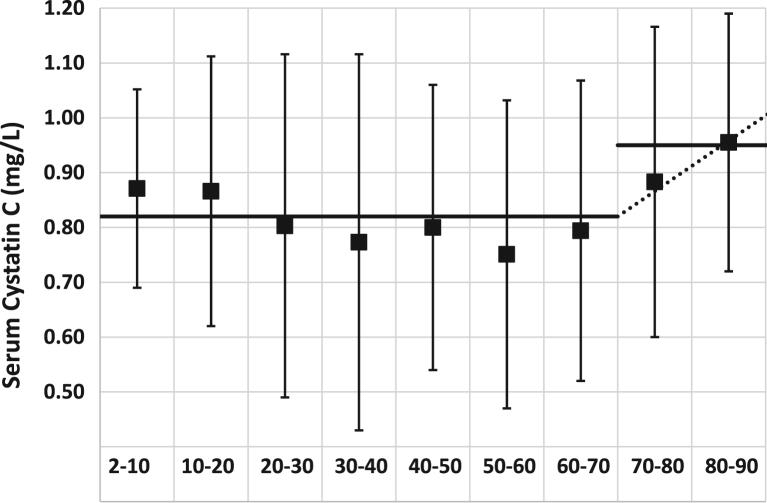
: Mean and reference intervals for serum cystatin C (mg/L) for age decades (years). The solid horizontal line corresponds with the choice of the normalization factor, 0.82 up to 70 years and 0.95 beyond 70 years of age. The vertical bars represent the interval from 2.5th Percentile (Pct) to 97.5th Pct as obtained from the Gaussian distribution for each decade.

For each decade, a truncated cumulative Gaussian fit was performed to determine the mean and standard deviation of the sample (Fig. 1 and Table 1). The dotted line in Fig. 1 represents the linear increase in normalization factor beyond the age of 70 years. In the FAS-cystatin C article [2] it was shown that there was no added value to using this (dotted) straight line fit for the normalization factor beyond 70 years, therefore, to keep it simple, the value of 0.95 mg/L was chosen as the rescaling factor for ScysC for ages > 70 years.

### 1.2 Rescaled biomarkers

The FAS-equation has been designed for Scr/Q_crea_ but it has recently been shown that it can also be used for ScysC/Q_cysC_ and for the combination of both normalized biomarkers [2], [5]. The fact that the same equation can be used to estimate mGFR from renal biomarkers also means that it is expected that Scr/Q_crea_ ≈ ScysC/Q_cysC_.

**Fig. 2:**
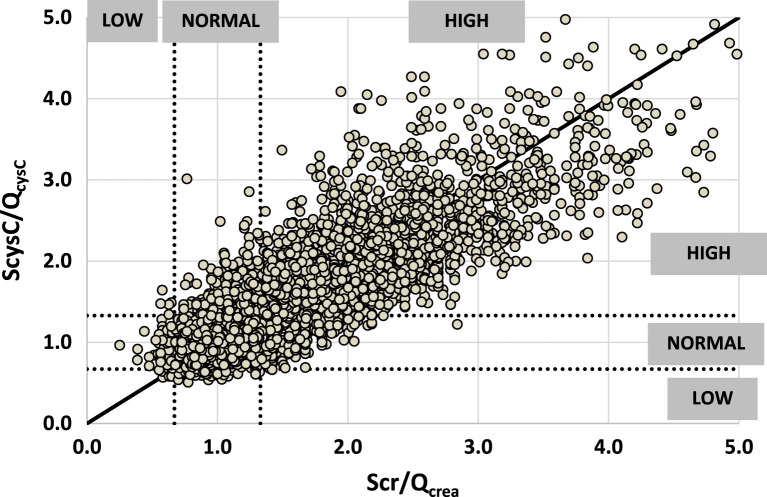
: Rescaled biomarker ScysC/Q_cysC_ against Scr/Q_crea_ for n=8584 subjects. The diagonal line is the identity line. The vertical and horizontal dotted lines correspond to ScysC/Q_cysC_ and Scr/Q_crea_ equal to 0.67 and 1.33 respectively and define the area of ‘normal’ biomarkers. Rescaled biomarker values < 0.67 are ‘Low’ and > 1.33 are indicated as ‘high’.

Fig. 2 is a scatterplot of ScysC/Q_cysC_ against Scr/Q_crea_, using the corresponding age/sex dependent Q_crea_-values and Q_cysC_-values, for all 8584 subjects. The diagonal line is the identity line, representing equal rescaled biomarkers. The scatter around the identity line indicates the amount to which the rescaled biomarkers deviate from each other. The overall Pearson correlation coefficient (r) between the rescaled biomarkers is 0.87 (p < 0.0001, n = 8584) and Lin's Concordance Correlation Coefficient is 0.857 with 95%CI [0.852–0.863]. Lin's CCC evaluates the degree to which pairs of observations fall on the diagonal or identity line through the origin. For children, r = 0.85, Lin's CCC = 0.828 (n = 767); for adults, r = 0.87 and Lin's CCC = 0.861 (n = 6068) and for older adults r = 0.88, Lin's CCC = 0.852 (n = 1749).

### 1.3 diagnostic value of the single rescaled biomarkers

The diagnostic value of the single renal biomarkers is presented in the Table 2a, Table 3a. The fixed threshold for mGFR of 60 mL/min/1.73 m² is compared to the age-dependent threshold CO_AD_ = 107.3/1.33 [ × 0.988^(Age-40)^ if Age > 40 years] [1], [6].

**Table 2a:** Frequency of patients with rescaled Serum creatinine ≤ and > 1.33 in the subgroups defined by mGFR (fixed and age-dependent threshold CO_AD_).

	Scr/Q_crea_ ≤ 1.33					Scr/Q_crea_ > 1.33					
Age Group	mGFR< 60	mGFR≥ 60	mGFR<CO_AD_	mGFR≥CO_AD_	Total	mGFR< 60	mGFR≥ 60	mGFR<CO_AD_	mGFR≥CO_AD_	Total	Grand Total
[2–10[	0	170	20	150	170	28	48	61	15	76	246
[10–20[	6	352	68	290	358	147	94	215	26	241	599
[20–30[	4	122	19	107	126	72	29	85	16	101	227
[30–40[	1	293	27	267	294	151	94	205	40	245	539
[40–50[	17	432	70	379	449	227	125	297	55	352	801
[50–60[	61	1543	105	1499	1604	385	142	441	86	527	2131
[60–70[	103	1317	111	1309	1420	683	168	681	170	851	2271
[70–80[	139	528	57	610	667	554	64	480	138	618	1285
[80–90[	96	147	23	220	243	180	10	153	37	190	433
≥ 90	17	3	5	15	20	32	0	27	5	32	52
	444	4907	505	4846	5351	2459	774	2645	588	3233	8584

**Table 2b:** Frequency of patients with rescaled Serum cystatin C ≤ and > 1.33 in the subgroups defined by mGFR (fixed and age-dependent threshold CO_AD_).

	ScysC/Q_cysC_ ≥ 1.33					ScysC/Q_cysC_ > 1.33					
Age Group	mGFR< 60	mGFR≥ 60	mGFR<CO_AD_	mGFR≥CO_AD_	Total	mGFR< 60	mGFR≥ 60	mGFR<CO_AD_	mGFR≥CO_AD_	Total	Grand Total
[2–10[	1	157	20	138	158	27	61	61	27	88	246
[10–20[	5	285	39	251	290	148	161	244	65	309	599
[20–30[	4	133	24	113	137	72	18	80	10	90	227
[30–40[	7	352	62	297	359	145	35	170	10	180	539
[40–50[	22	500	103	419	522	222	57	264	15	279	801
[50–60[	53	1595	110	1538	1648	393	90	436	47	483	2131
[60–70[	113	1352	122	1343	1465	673	133	670	136	806	2271
[70–80[	229	570	103	696	799	464	22	434	52	486	1285
[80–90[	94	152	22	224	246	182	5	154	33	187	433
≥ 90	14	3	3	14	17	35	0	29	6	35	52
	542	5099	608	5033	5641	2361	582	2542	401	2943	8584

#### 1.3.1 Serum creatinine

**Fig. 3:**
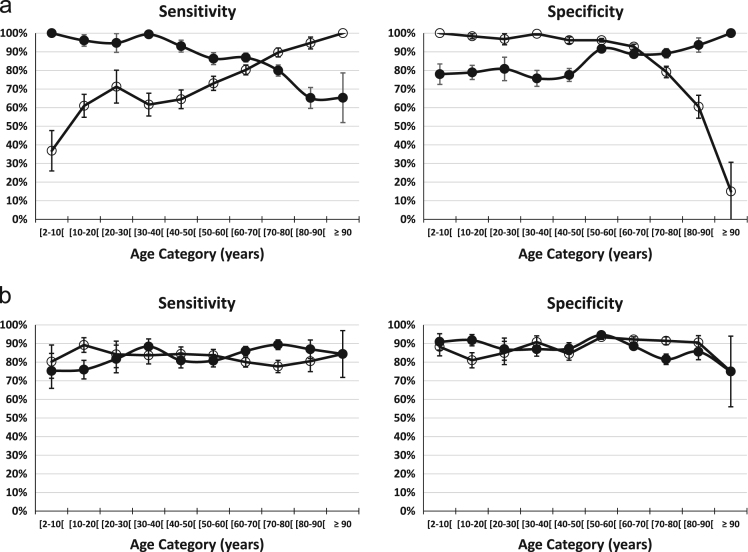
**a**: Sensitivity and Specificity per age-category. Solid circles correspond to Scr/Q_crea_ as the test result (positive when > 1.33, negative when ≤ 1.33) and diseases status defined by the fixed mGFR threshold of 60 mL/min/1.73 m². Open circles correspond to the reversed situation, that is, mGFR as the test result (positive when mGFR < 60 and negative when mGFR ≥ 60) and disease status defined by the Scr/Q_crea_ threshold of 1.33. **b**: Sensitivity and Specificity per age-category. Solid circles correspond to Scr/Q_crea_ as the test result (positive when > 1.33, negative when ≤ 1.33) and diseases status defined by the age-dependent mGFR threshold CO_AD_. Open circles correspond to the reversed situation, that is, mGFR as the test result (positive when mGFR < CO_AD_ and negative when mGFR ≥ CO_AD_) and disease status defined by the Scr/Q_crea_ threshold of 1.33.

Sensitivity (S) and Specificity (Sp) in Fig. 3a-b are calculated as follows:

a) in case a true positive test result is defined as Scr/Q_crea_ > 1.33 in the mGFR < 60 subgroup, and a true negative test result is defined as Scr/Q_crea_ ≤ 1.33 in the mGFR ≥ 60 subgroup. E.g. in the age-group 2–10 years, S = 28 / (28 + 0) = 100% and Sp = 170 / (170 + 48) = 78.0%; in the age-group 80–90 years, S = 180 / (180 + 96) = 65.2% and Sp = 147 / (147 + 10) = 93.6%. Reversing the role of Scr/Q_crea_ and mGFR, we find for the 2–10 year age-group: S = 28/76 = 36.8% and Sp = 170/170 = 100%; in the age-group 80–90 years, we have S = 180/190 = 94.7% and Sp = 147/243 = 60.5%.
b) in case a true positive test result is defined as Scr/Q_crea_ > 1.33 in the mGFR < CO_AD_ subgroup, and a true negative test result is defined as Scr/Q_crea_ ≤ 1.33 in the mGFR ≥ CO_AD_ subgroup. E.g. in the age-group 2–10 years, S = 61 / (61 + 20) = 75.3% and Sp = 220 / (220 + 37) = 85.6%; in the age-group 80–90 years, S = 180 / (180 + 96) = 65.2% and Sp = 147 / (147 + 10) = 93.6%. Reversing the role of Scr/Q_crea_ and mGFR, we find for the 2–10 year age-group: S = 61/76 = 80.3% and Sp = 150/170 = 88.2%; in the age-group 80–90 years, we have S = 153/190 = 80.5% and Sp = 220/243 = 90.5%.

#### 1.3.2 Serum cystatin C

**Fig. 4:**
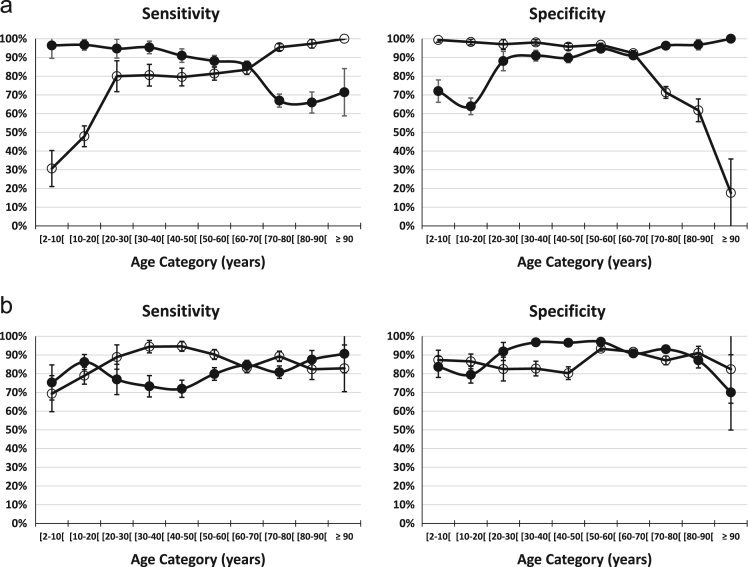
**a**: Sensitivity and Specificity per age-category. Solid circles correspond to ScysC/Q_cysC_ as the test result (positive when > 1.33, negative when ≤ 1.33) and disease status defined by the fixed mGFR threshold of 60 mL/min/1.73 m². Open circles correspond to the reversed situation, that is, mGFR as the test result (positive when mGFR < 60 and negative when mGFR ≥ 60) and disease status defined by the ScysC/Q_cysC_ threshold of 1.33. **b**: Sensitivity and Specificity per age-category. Solid circles correspond to ScysC/Q_cysC_ as the test result (positive when > 1.33, negative when ≤ 1.33) and diseases status defined by the age-dependent mGFR threshold CO_AD_. Open circles correspond to the reversed situation, that is, mGFR as the test result (positive when mGFR < CO_AD_ and negative when mGFR ≥ CO_AD_) and disease status defined by the ScysC/Q_cysC_ threshold of 1.33.

Sensitivity (S) and Specificity (Sp) are calculated as follows:

a) in case a true positive test result is defined as ScysC/Q_cysC_ > 1.33 in the mGFR < 60 subgroup, and a true negative test result is defined as ScysC/Q_cysC_ ≤ 1.33 in the mGFR ≥ 60 subgroup. E.g. in the age-group 2–10 years, S = 27 / (27 + 1) = 96.4% and Sp = 157 / (157 + 61) = 72.0%; in the age-group 80–90 years, S = 182 / (182 + 94) = 65.9% and Sp = 152 / (152 + 5) = 96.8%. Reversing the role of ScysC/Q_cysC_ and mGFR, we find for the 2–10 year age-group: S = 27/88 = 30.7% and Sp = 285/290 = 98.3%; in the age-group 80–90 years, we have S = 182/187 = 97.3% and Sp = 152/246 = 61.8%.
b) in case a true positive test result is defined as ScysC/Q_cysC_ > 1.33 in the mGFR < CO_AD_ subgroup, and a true negative test result is defined as ScysC/Q_cysC_ ≤ 1.33 in the mGFR ≥ CO_AD_ subgroup. E.g. in the age-group 2–10 years, S = 61 / (61 + 20) = 75.3% and Sp = 138 / (138 + 27) = 83.6%; in the age-group 80–90 years, S = 154 / (154 + 22) = 87.5% and Sp = 224 / (224 + 33) = 87.2%. Reversing the role of ScysC/Q_cysC_ and mGFR, we find for the 2–10 year age-group: S = 61/88 = 69.3% and Sp = 138/158 = 87.3%; in the age-group 80–90 years, we have S = 154/187 = 82.4% and Sp = 224/246 = 91.1% (Fig. 4).

### 1.4 Interchangeability between biomarkers and mGFR / FAS-eGFR

Comparing (Scr/Q_crea_+ScysC/Q_cysC_)/2 using the threshold of 1.33 with mGFR using the fixed threshold of 60 mL/min/1.73 m², for the complete n = 8584 dataset, to detect renal impairment, we have (Table 3a):

**Table 3a:** 2×2 frequency table comparing measured GFR (with fixed threshold of 60 mL/min/1.73 m²) with the average of the biomarkers (with threshold 1.33).

		mGFR		
		**≥ 60**	**< 60**	**Total**
**Average of Biomarkers**	**≤ 1.33**	5067	415	5482
	**> 1.33**	614	2488	3102
	**Total**	5681	2903	8584

Exact McNemar's test: p < 0.0001. % agreement = (5067 + 2488) / 8584 = 88.0%.

Comparing (Scr/Q_crea_+ScysC/Q_cysC_)/2 using the threshold of 1.33 with mGFR using an age-dependent threshold, for the complete n = 8584 dataset, to detect renal impairment, we have (Table 3b):

**Table 3b:** 2×2 frequency table comparing measured GFR (with age-dependent threshold) with the average of the biomarkers (with threshold 1.33).

		mGFR		
		**≥ CO**_**AD**_	**< CO**_**AD**_	**Total**
**Average of Biomarkers**	**≤ 1.33**	5043	439	5482
	**> 1.33**	391	2711	3102
	**Total**	5434	3150	8584

Exact McNemar's test: p = 0.1027. % agreement = (5043 + 2711) / 8584 = 90.3%.

Using the FAS_combi_ equation to calculate eGFR from both Scr/Q_crea_ and ScysC/Q_cysC_, the following table is obtained when comparing FAS-eGFR using the age-dependent threshold with the combined biomarker value (Scr/Q_crea_+ScysC/Q_cysC_)/2 using the threshold of 1.33 (Table 4):

**Table 4:** 2×2 frequency table comparing (FAS) estimated GFR (with age-dependent threshold) with the average of the biomarkers (with threshold 1.33).

		FAS-eGFR		
		**≥ CO**_**AD**_	**< CO**_**AD**_	**Total**
**Average of Biomarkers**	**≤ 1.33**	5482	0	5482
	**> 1.33**	0	3102	3102
	**Total**	5482	3102	8584

**Fig. 5:**
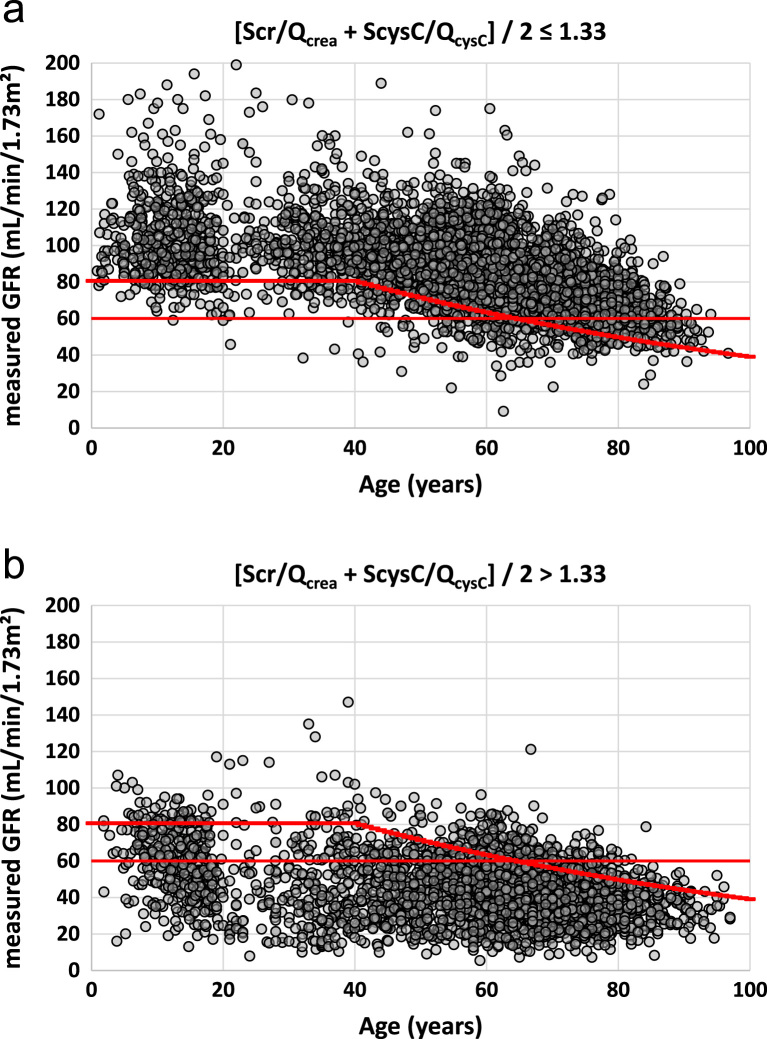
**a-b**. Measured GFR against age for n = 5482 subjects with the mean of both biomarkers ≤1.33 (top), and n = 3102 with the mean of both biomarkers >1.33 (bottom). The horizontal red line is the fixed GFR-threshold of 60 mL/min/1.73 m² and the curved red line is the age-dependent threshold CO_AD_.

In Fig. 5a-b, the raw mGFR-values are plotted against age, for the subgroups defined by (Scr/Q_crea_+ScysC/Q_cysC_)/2 below and above the threshold of 1.33, together with the fixed threshold for mGFR = 60 mL/min/1.73 m² and the age-dependent threshold obtained from the FAS-equation with (Scr/Q_crea_+ScysC/Q_cysC_)/2 = 1.33. These figures correspond to the Table 3a, Table 3b.

## 2 Experimental design, materials and methods

This is a retrospective study, where the data presented here were collected from 12 previously published cohorts (grand total of 8584 patients) and centralized for pooled data-analysis. Assay data for Scr and ScysC, together with measured GFR, age, sex were centralized for the data-analysis. The total number of patients was subdivided into subgroups corresponding with age-decades with the aim to perform a data-analysis of the diagnostic value (in terms of sensitivity and specificity) of the biomarkers per age-decade. Sensitivity and specificity were calculated with reference to measured GFR (fixed and age-dependent threshold), and with reference to the rescaled biomarker threshold of 1.33.

Scr was traceable to the gold standard Isotope Dilution Mass Spectrometry method, ScysC was obtained from assays calibrated to the international standard or ScysC was recalculated against the calibrator and measured GFR was obtained from accepted reference methods, as described in the main article [1].
